# Predominately Uncultured Microbes as Sources of Bioactive Agents

**DOI:** 10.3389/fmicb.2016.01832

**Published:** 2016-11-18

**Authors:** David J. Newman

**Affiliations:** Newman Consulting LLCWayne, PA, USA

**Keywords:** natural product sources, uncultured microbes, poor producing microbes, endophytes, microbe–microbe interactions

## Abstract

In this short review, I am discussing the relatively recent awareness of the role of symbionts in plant, marine-invertebrates and fungal areas. It is now quite obvious that in marine-invertebrates, a majority of compounds found are from either as yet unculturable or poorly culturable microbes, and techniques involving “state of the art” genomic analyses and subsequent computerized analyses are required to investigate these interactions. In the plant kingdom evidence is amassing that endophytes (mainly fungal in nature) are heavily involved in secondary metabolite production and that mimicking the microbial interactions of fermentable microbes leads to involvement of previously unrecognized gene clusters (cryptic clusters is one name used), that when activated, produce previously unknown bioactive molecules.

## Introduction

Over the past twenty-plus years, data related to microbes from all sources being involved in the production of bioactive agents, has grown from being a “suggestion or slight possibility” to now being recognized, at least in the marine area, as probably being the source(s) of the majority of bioactive agents that have been reported from marine invertebrates. However, in the vast majority of cases, the microbe(s) cannot be cultivated under the usual conditions that had been used for many years. The advances in genomic analysis techniques, particularly in the last five or so years, has however, permitted the amplification and then the subsequent analysis from a biosynthetic perspective, of the genes within a single “as yet uncultured” microbe, and thus the identification of the “source” of the bioactive materials reported from the “host.”

In the case of plant-derived bioactive compounds, what has now occurred with regularity, are reports of mainly endophytic microbes, usually fungi, but frequently actinobacteria, that will produce low levels of the “plant metabolite” on fermentation but on subsequent sub-culturing, the microbe apparently loses its ability to produce the “plant-metabolite.” However, in the last few years, investigators have begun to “relearn” techniques that used to be very common in the pharmaceutical industry, but to the ultimate chagrin of academic researchers, were never published in regular journals, that supplementation of fermentation broths with extracts of parts of the “nominal producing source,” could “induce or maintain” production of the metabolite of interest.

The other component of “production” that has now come to the fore, is the belated recognition that in any microbial system, there is constant chemical messaging between microbes in the microbiome of the host. Whereas microbiologists and co-workers want a single microbe to produce a given compound, Mother Nature prefers a consortium and thus the recognition that mixed cultures might well aid in “production” has now become quite apparent, usually via the “switching on or off” of biosynthetic gene clusters (BGCs) in one or more of the consortium.

This short review will cover various aspects of the above, not in depth but relevant up to date references will be provided so that the reader can further investigate the areas discussed.

## Marine-Sourced Bioactive Agents and Microbes

### The Naphthyridinomycin/Tetrahydroisoquinoline Derivatives

**FIGURE 1:**
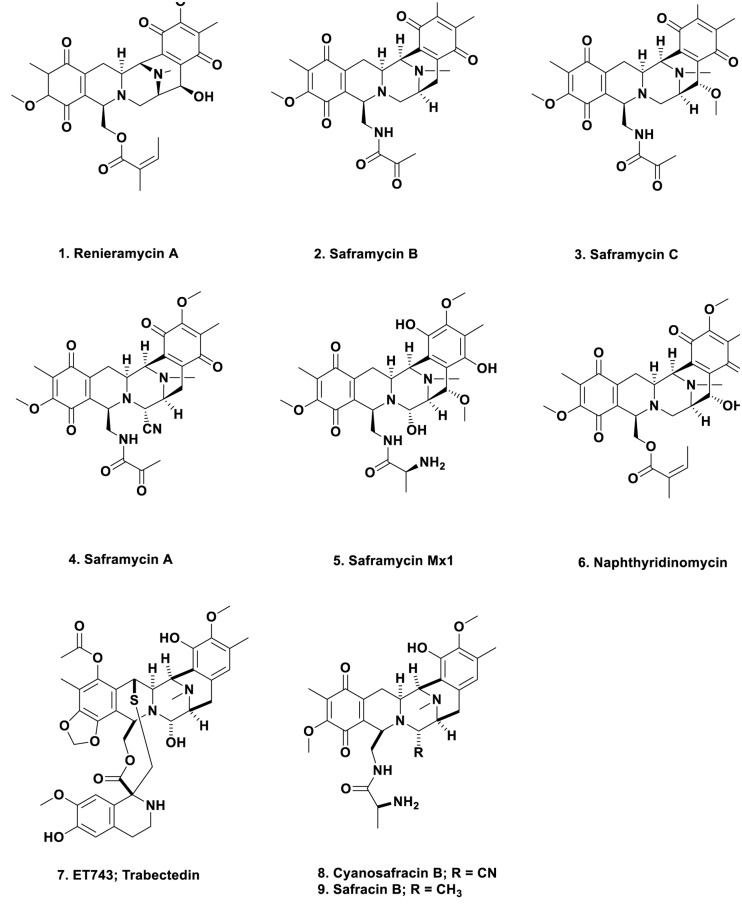
**Compounds 1 to 9**.

**FIGURE 2:**
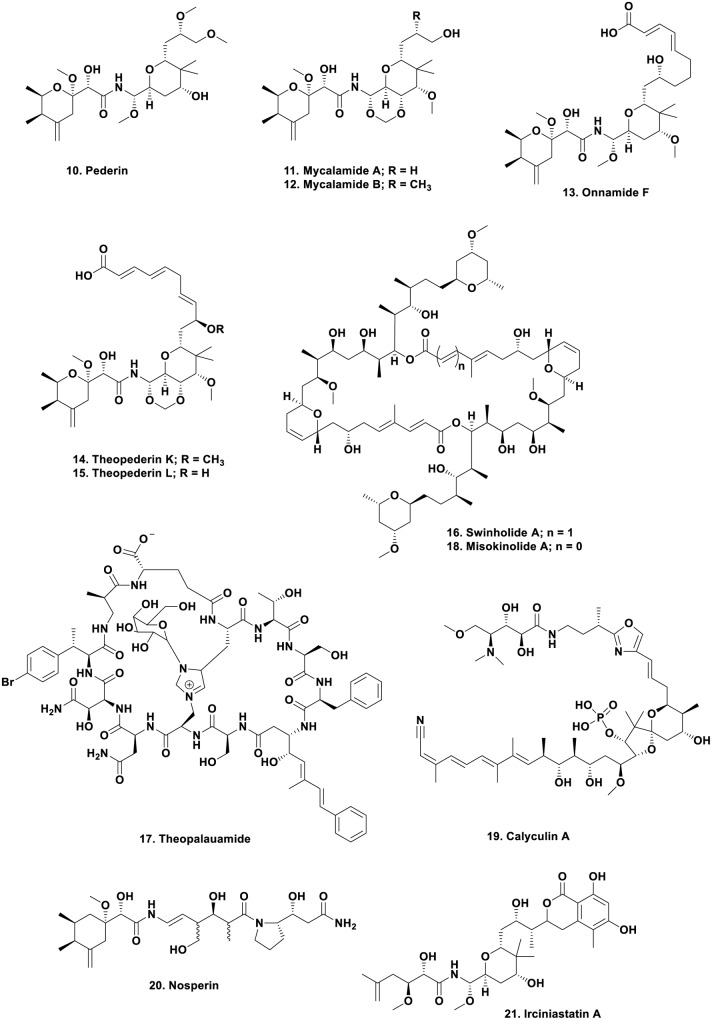
**Compounds 10 to 21**.

**FIGURE 3:**
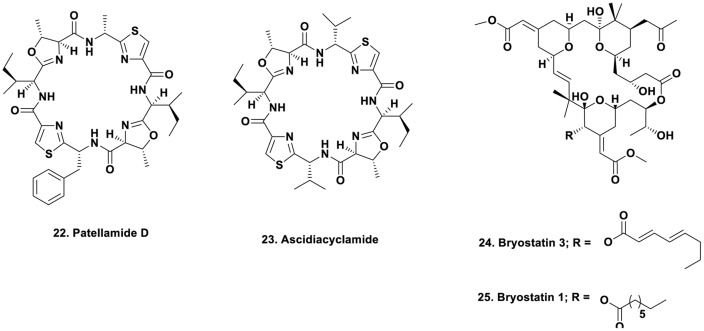
**Compounds 22 to 25**.

**FIGURE 4:**
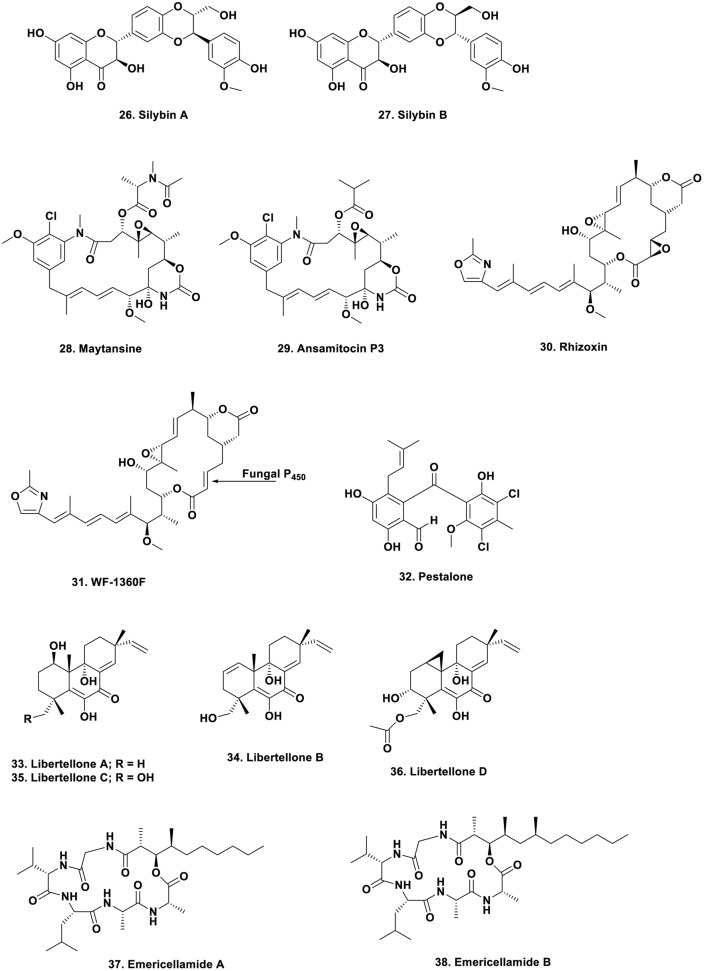
**Compounds 26 to 38**.

In 1982, the Faulkner group at the Scripps Institute of Oceanography reported the isolation of renieramycin A (**Figure 1**; 1) from the Eastern pacific sponge of the genus *Reniera* sp. (Frincke and Faulkner, 1982). This material had antibiotic properties and its structure was very similar to the known antitumor agents of the saframycin class that had been reported 5 years earlier by Takahashi and Kubo (1977), from the terrestrial microbe, *S. lavendulae.* Two later papers (Arai et al., 1979, 1980) gave the structures of saframycins B (**Figure 1**; 2) and C (**Figure 1**; 3), and then of saframycin A, (**Figure 1**; 4), respectively. These reports were then followed in 1988 by the isolation of saframycin Mx 1 (**Figure 1**; 5) from the myxobacterium *Myxococcus xanthus* strain Mx48 (Irschik et al., 1988). Thus in just over 10 years, closely related antibacterial and antitumor compounds had been isolated from a streptomycete, a myxobacterium (both terrestrial) and from a marine sponge. However, these were only the tip of the iceberg, as the base molecule, naphthyridinomycin, (**Figure 1**; 6) was initially reported from Canada in 1974 (Sygusch et al., 1974) and 1975 (Kluepfel et al., 1975), being isolated from the terrestrial streptomycete *Streptomyces lusitanus* AY B-1026.

***Why is this early work so important from a marine perspective?*** In the middle 1980s to early 1990s, the Rinehart group at the University of Illinois at Urbana, the second group after the Scheuer group in Hawaii to systematically study marine-derived compounds, and then later, in conjunction with the Wright group at Harbor Branch Oceanographic Institution in Florida, published two back to back papers in the Journal of Organic Chemistry showing the structures of the cytotoxic agent ET743 (**Figure 1**; 7) and its congeners, isolated from the Caribbean tunicate, *Ecteinascidia turbinata* (Rinehart et al., 1990; Wright et al., 1990). These reports were an extension of the work reported by Holt (1986) in his Ph.D. thesis completed while in the Rinehart group. That this organism “produced” a cytotoxic compound or compounds had been reported in 1969 by Sigel et al. (1970). These compounds were obviously built on the same basic structure as reported for naphthyridinomycin, the saframycins, and renieramycin. Thus one now had multiple bioactive compounds that must have been produced by a similar set of biosynthetic clusters, though it was unknown at the time what the organism or organisms might be, but microbes were prime candidates.

ET743 became an approved antitumor drug under the aegis of the Spanish company PharmaMar and the methods used in its production included: massive large-scale collections; aquaculture of the tunicate in sea and in lakes; then partial synthesis using a marine bacterial product, cyanosafracin B (**Figure 1**; 8) to produce cGMP ET743. The story leading to the production of ET743 has been presented by a number of people, but the best publications are those from the PharmaMar team (Cuevas et al., 2000, 2012; Menchaca et al., 2003; Cuevas and Francesch, 2009).

In addition to the publications from the PharmaMar group on the semisynthetic processes, two other highly relevant reviews are the one in 2002 by Scott and Williams covering the chemistry and biology of the tetrahydroquinoline antibiotics (Scott and Williams, 2002), which was followed in 2015 by a very thorough review on the ecteinascidins themselves in 2015, again from the Williams group (Le et al., 2015).

From a microbial aspect there were suggestions that an as yet uncultured bacterium, *Candidatus Endoecteinacidia frumentenis* (AY054370), was involved in the production of these molecules. This organism was found in ET743-producing *E. turbinata* collected in both the Caribbean and the Mediterranean seas (Moss et al., 2003; Perez-Matos et al., 2007). By use of the suggestions made by Piel (2006) as to how to utilize symbionts from invertebrates, and then using the knowledge of the organization of the BGCs of the saframycins (Li et al., 2008) and safracin B (**Figure 1**; 9) (Velasco et al., 2005) as markers, the Sherman group at the University of Michigan, were able to identify the “contig” that encoded the NRPS biosynthetic enzymes involved in the ET743 complex, as well as the probable producing bacterium, the as yet uncultured microbe *Candidatus Endoecteinascidia frumentensis* present in both the Caribbean and Mediterranean *E. turbinata* organisms (Rath et al., 2011). Four years later, the same group directly confirmed the initial report (Schofield et al., 2015). In the process, they demonstrated that the producing bacterium, *E. frumentensis*, may well represent a member of a new family of *Gammaproteobacteria* and has an extensively streamlined genome as found in other symbiotic microbes (McCutcheon and Moran, 2012), with most of the genetic machinery being devoted to this complex of compounds (Kehr and Dittmann, 2015).

## Marine Metabolites Based Upon a Terrestrial Beetle Toxin

### Mycalamides, Onnamides, and Similar Molecules

The structure of the toxin pederin (**Figure 2**; 10) “used” by rove beetles of the genus *Paederus* as a protective agent, was first identified chemically by Italian scientists studying this genus in a publication in 1952 (Pavan and Bo, 1952). The dermatitis caused by the toxin has been well described in the literature, with a recent publication by Cressey et al. (2013) demonstrating the problem with this toxin.

Following on from the original 1952 publication, in 1965 Carani et al. (Cardani et al., 1965) published an initial structure which was then revised 3 years later by Matsumoto et al. (1968) giving the structure shown (**Figure 2**; 10). This is where the story might have languished with the compound simply becoming an interesting molecule to synthesize by demonstrating novel chemical methods. Such syntheses have been reported in some relatively recent publications in the synthetic chemistry literature (Jewett and Rawal, 2007; Wu et al., 2011). However, in the late 1980s, the Blunt and Munro group at the University of Canterbury in New Zealand, published their finding that an extract of a relatively deep water sponge of the genus *Mycale*, collected in cold water, produced bioactive molecules that had antiviral and cytotoxic biological activities, and encompassed the pederin nucleus within the overall structure. The molecules were also powerful vesicants, as is pederin. The two molecules were named mycalamide A (**Figure 2**; 11) and B (**Figure 2**; 12) differing only by a methyl group, but with a 10-fold difference in biological activity (Perry et al., 1988, 1990).

***What is the relationship of such findings to pederin and Paederus beetles?*** The following reports in the insect physiology literature will help uncover the reasons why this was a very important finding, that led, many years later, to the identification of the actual “source” of the pederin-related molecules found in the *Mycale* sp., and in other sponges, in particular, the *Theonella swinhoei* “Yellow variant.” In 1999, a German entomologist, Rupert Kellner, published a very interesting paper that asked; “what was the basis of pederin polymorphism in the rove beetle *Paederus riparius*?” His suggestion, with data, was that an endosymbiont was the actual producer of the toxin (Kellner, 1999). Then 2 years later, he reported that pederin biosynthesis was suppressed in the closely related species, *Paederus sabaeus*, if antibiotics were used to remove endosymbionts (Kellner, 2001). Thus there was significant evidence implying a bacterial component in the production of pederin, and since this occurred in two different species of the beetle, it might be common to all.

However, to bring the story to its climax, one now has to return to the marine environment. From 1988 to early 2000, there were reports that a significant number of sponge extracts contained pederin-related molecules such as the onnamides, with onnamide F (**Figure 2**; 13) being a good example (Vuong et al., 2001), together with the theopederins, with compounds such as theopederins K (**Figure 2**; 14) and L (**Figure 2**; 15) being examples of the structural similarities (Paul et al., 2002). Kellner (2002b) reported that he had identified the endosymbiont as a very close relative to *Pseudomonas aeruginosa* and showed that interspecific transmission of the endosymbionts was related to the different genetic makeup of individual isolates (Kellner, 2002a).

***So how did this work then relate to the “marine-derived pederin-like compounds”?*** In a series of papers in the time frame from 2002 to 2005, Piel demonstrated that he could find the biosynthetic clusters for pederin in the putative pseudomonad identified and isolated by Kellner (Piel, 2002), and that these had an unprecedented diversity of catalytic domains in the first four clusters in the process (Piel et al., 2004c). Since Piel had the relevant genetic probes, he collaborated with the Japanese group led by Fusetani and Matsunaga at the University of Tokyo, to investigate the production of the closely related onnamides, isolated from the Japanese sponge *Theonella swinhoei* (yellow variant) in warm, shallow waters off of Okinawa, in contrast to the mycalamide-producer. The pseudomonal probes “lit up” parts of the sponge metagenome, and they were able to locate the nexus of the biosynthesis to an as yet uncultivated symbiont (Piel et al., 2004b). Concomitantly Piel also demonstrated evidence for what is now known as a “symbiosis island” that permitted horizontal acquisition of the pederin biosynthetic capabilities in *Paederus fuscipes* (Piel et al., 2004a). The details as of that time were published in a short review in the Journal of Natural Products in 2005 (Piel et al., 2005), and then in 2011, Kador et al. (2011) published separate oligonucleotide probes that could be used to detect pederin producers in *Paederus* beetles.

Following on from these seminal studies, Wilson and Piel (2013) demonstrated potential approaches to the study of uncultivated, or not yet cultivatable microbes, as resources for novel biosynthetic enzymology. This paper demonstrated the potential for performing genomic work on very small numbers of uncultivated bacteria isolated from invertebrate hosts, in this case, sponges, and tunicates. This was exactly the type of investigation that Bewley and Faulkner (1998) wished to perform in the late 1990s when they identified the production of the cyclic peptides swinholide A (**Figure 2**; 16) and theopalauamide (**Figure 2**; 17) by *Theonella swinhoei*, and their suggestion, using the techniques then available (microscopy in general), that a microbial consortium might be responsible for their “production.”

In 2014, 1 year after their earlier paper, the Piel group demonstrated in a seminal publication in Nature, that the producing organism in *T. swinhoei* was an as yet uncultured microbe, and that there were two subtly distinct variations isolated, TSY-1 and TSY-2 via single cell separation of the sponge and contents. Both contained a plasmid that contained the onnamide and polytheonamide BGCs, but further investigation showed that they differed significantly when BGC clusters that were chromosomally encoded were studied. Thus the TSY-1 variant in addition to the plasmid-encoded BGCs, also contained the genes for a further 28 BGCs including cyclotheonamides, proteusins, and ceramides, plus others. The other strain, TSY-2 only had seven other BGCs with very little overlap with TSY-1 metabolites. As a result of these and other studies on different sponge taxa, the suggestion was made that these were representative of a new phylum “Tectomicrobia.” Thus these two microbes contained the necessary genetic machinery to produce 31 of the then 32 known cytotoxins to have been isolated from this particular sponge at that time (Wilson et al., 2014).

Recently, the Piel group have published the enzymology involved in the formation of the long peptides such as the polytheonamides which have repeating D and L amino-acids, but are ribosomally produced peptides (Morinaka et al., 2014), with a recent publication by Hayata et al. (2016) demonstrating syntheses around this structure to produce new ion-channel cytotoxins. Morionka et al. (2014), was then followed in 2016 by a review demonstrating the metabolic potential of the as yet uncultivated “*Entotheonella*” where more information is given as to the multiplicity of structures that result from this microbe (Freeman et al., 2016).

Looking at an area where many marine-derived bioactive compounds have been reported, but none have yet made into clinical trials, Ueoka et al. (2015) published an extensive paper on the source of misokinolide A, a compound that differs from the well-known swinholide A by removal of one double bond in the ring structure (**Figure 2**; 18), demonstrating that it too, came from a non-cultivated *Entotheonella* but not from *T. swinhoei* but from a *Discodermia* species. Interestingly the protein phosphatase inhibitor calyculin A (**Figure 2**;19) produced by *Discodermia calyx*, was shown by Wakimoto et al. (2014) to be a product of the same microbial genus, then in a more thorough paper published in 2016, they demonstrated that it is actually produced as a pro-drug (Wakimoto et al., 2016). Such a pro-drug approach may well be a protection method to avoid killing the host invertebrate or even the producing microbe.

To conclude the microbial chemistry aspect of this section, but now moving back to the terrestrial sphere, there was a very intriguing report showing the presence of a pederin-like compound, nosperin (**Figure 2**; 20) found in a lichen, where the bacterium was a cyanobacterium, a *Nostoc* sp., so these biosynthetic genes are extremely widespread (Kampa et al., 2013).

As mentioned earlier, the structures of pederin and its derivatives certainly excited synthetic organic chemists and over the years, effectively all of the molecules that contain the pederin backbone have been synthesized, even when ring-opened as in irciniastatin A (**Figure 2**; 21). This compound was initially reported by the Pettit group in 2004 (Pettit et al., 2004) and subsequently reported as psymberin by the Crews group the same year (Cichewicz et al., 2004) from a different sponge genus. Careful inspection of the supporting information in the Crews paper showed that they knew of the same compound under a different name/genus from the Pettit group, published before their submission. Thus the Pettit group has priority in the discovery of this compound structure. Representative examples of these compounds have been synthesized by considerable numbers of organic chemists in the last 10 plus years. The following papers should be consulted by readers interested in the synthetic processes used (Jewett and Rawal, 2007; Jiang et al., 2007; Nishii et al., 2009; Wan et al., 2011; Wu et al., 2011, 2012; Mosey and Floreancig, 2012; Floreancig, 2014; Uesugi et al., 2015). Of these, the review by Mosey and Floreancig (2012) gives a relatively thorough overview of the isolation, biological activities, and medicinal chemistry of these agents.

Thus what began as a discussion of the toxin produced by the blister beetle that was known in Brazilian forests/jungle and in other parts of the World, led to the ability to identify and express genetic loci related to the biosynthesis of the agent, but then “migrated” into areas not even thought to be possible; that the beetle toxin was in fact, used by biodiversity (aka Mother Nature) to generate molecules in organisms as diverse as shallow and deep water marine sponges, in warm (close to 35°C) and cold (2°C) water environments and even in terrestrial lichens. None of these were even thought of in the wildest dreams of the original scientists working on beetle toxins.

### Combinatorial Chemistry in Ascidians

In the encrusting ascidians such as *Lissoclinum* species, investigators over the years have found a series of bioactive agents mainly based on cyclic peptides. For many years, the actual source of the agents, in particular the patellamides, was not known. In a presentation at a Society of Industrial Microbiology Meeting very late in 2004, Long et al. (2005) presented evidence that the probable producer was an as yet uncultivated cyanophyte, a “*Prochloron”* species. Unfortunately, this paper was not included in the printed list of papers presented (only discovered after the presentation at the meeting), thus in the absence of a dated presentation, the absolute priority to this group could not be formally assigned. Long et al. (2005) demonstrating that the complete *Prochloron* sp. genome could be moved into an *E. coli* host using shotgun cloning techniques. *Prochloron* samples were extracted from the cloacal cavities of ten specimens of *L. patella*, each showing a matching patellamide composition according to HPLC analysis of the holobiont extracts. Using bacterial artificial chromosome techniques and then attempting to express the patellamide producing genes using primers failed. However, by use of standard fermentation techniques, clones were obtained that produced patellamide D **Figure 3**; 22) and ascidiacylamide (**Figure 3**; 23).

Whilst this paper was in press, the Schmidt group published similar results using a different *L. patella* source from that used by Long et al. (2005; Schmidt et al., 2005). The Schmidt group used material from the Republic of Palau instead of the Great Barrier Reef (Long et al., 2005) and identified the relevant gene clusters in the isolated *Prochloron* species. Since they had been working on a full genomic analysis of this symbiont, they had information available pointing to potential gene biosynthetic clusters, enabling a rapid assessment of the potential biosynthetic clusters by expressing them in *E. coli*. Thus two independent groups came up with the same source of the patellamides.

The Schmidt group then proceeded over the next few years to further analyses of the producing symbiont, uncovering a fascinating series of complex interactions that permitted substitution of individual aminoacids in these ribosomally produced products, that had geographic relationships defined in terms of what mixture of patellamides and the closely related trunkamides were produced by individual ascidians. For the interested reader, a significant number of publications related to this symbiotic relationship have been published by the Schmidt group and their collaborators. These should be perused in detail to demonstrate the complexity of the interactions involved (Donia et al., 2011; Kwan et al., 2012, 2014; Schmidt et al., 2012; Tianero et al., 2012, 2015; Adnani et al., 2014; Schmidt, 2014, 2015). The most current paper is one published very recently in Applied and Environmental Microbiology demonstrating the intimate involvement of the microbe in this process (Lin et al., 2016).

### Source of Bryostatins in *Bugula neritina*

The bryozoan *B. neritina* first came to the attention of natural product chemists when Pettit reported the isolation of bryostatin 3 (**Figure 3**; 24), and then over the next few years, elucidated the structures of 18 bryostatins, all isolated from massive wild collections of *B. neritina* either in the Gulf of Florida, or other coastal areas of the US, with a massive collection in Palos Verdes, California that led to the isolation of gram quantities of bryostatin 1 by workers at NCI-Frederick (Schaufelberger et al., 1991). The story up through late 2011 was given by in a review by Newman published as a chapter in the second edition of “Anticancer Agents from Natural Products” in 2012 (Newman, 2012). Over 80 clinical trials of bryostatin 1 (**Figure 3**; 25) with or without other cytotoxic agents have been reported over the years at Phase I or Phase II levels against various cancers, but none have demonstrated activities that have warranted continuation. Currently there is one trial shown at the Phase II level in Alzheimer’s disease (NCT02431468) under the aegis of Neurotrope Inc.

The actual producing agent in *B. neritina* was unknown until the report by Haygood and Davidson (1997), demonstrating that the larvae of bryostatin-producing bryozoans contained a previously unknown bacterium that could not be cultured, but was observable in the pallial sinus of the larvae. This microbe was named as “*Candidatus Endobugula sertula*” by Haygood, and over the next few years, this microbe was found in other examples of *B. neritina* but appeared to have a latitudinal restriction and strain variation with depth. These phenomena were well investigated by both the Haygood group on the Pacific Coast, and by the Lopanik group in the Atlantic Coast of the USA. The relevant publications are (Davidson and Haygood, 1999; Davidson et al., 2001; Lopanik et al., 2004, 2006; Sharp et al., 2007; Linneman et al., 2014).

The role of bryostatin within the larvae appears to be a protective measure. This was shown by investigation of the effect of removing the symbiont via antibiotic treatment, followed by predation studies on larvae containing the symbiont, versus those without it. The levels of bryostatins were also measured in these experiments and they demonstrated that without bryostatin production, the larvae were “food” for predators (Lopanik et al., 2004). What was of immense import, however, was the work performed by the Sherman group at the University of Michigan, in conjunction with the Haygood and Lopanik groups, where they identified the putative bryostatin gene cluster from “*Candidatus Endobugula sertula*” in 2007 (Sudek et al., 2007). This initial paper was followed up by the Sherman group in 2008 (Lopanik et al., 2008) and 2010 (Buchholz et al., 2010), leading to a very recent paper demonstrating the “holobiont fitness” via specific interactions with host organism PKC enzymes from the Lopanik group (Mathew et al., 2016). Thus even if the organism’s symbiont cannot yet be cultivated, its effect and product can be measured by modern techniques.

Finally, a very interesting paper was published recently demonstrating that molecules based on the bryostatin skeleton can have significant effects on viral replication. Paul Wender at Stanford University, who for many years has been involved in modifying the basic structure of the bryostatins and other potent marine-derived biologically active natural products, producing bioactive truncated structures. In the case of the bryostatin-based compounds they are known colloquially as “bryologs,” and Wender et al. published a paper in 2016 demonstrating the inhibition of Chikungunya virus-induced cell death, by a relatively simple bryostatin analog, that does not appear to use a PKC pathway, in contrast to the usual mechanism of action of bryostatins (Staveness et al., 2016). Thus close to 50 years since the first reports of bryostatins, they are still an active structural class for synthetic chemists to modify.

## Plant-Sourced Compounds and Microbes

In a significant number of cases nowadays, there have been reports in the literature that have questioned the “actual source” of biologically active materials that are approved pharmaceuticals, or were leads to the approved drug entity. These are often, though not exclusively, antitumor agents in clinical use that were isolated from plants. Very few came from plants quoted to have some medicinal properties, with the majority found from bioactivity-directed isolation techniques under the funding and collection programs of the US National Cancer Institute. In contrast to the microbes referred to above, these “endophytes” are usually able to initially be fermented and do produce the “plant metabolite” albeit in very low yield. In general, on subsequent refermentation, the yields decreased and frequently after one or more sub-culturings, the compound was no longer observable. Thus initial reports were criticized for not accounting for “carry-over” of the active agent during the microbe’s isolation. This latter comment, and the inability to successfully repeat many fermentations with continued production of the metabolite, was seen over the years for “plant-derived” compounds such as taxol, camptothecin, the vinca alkaloids and others.

However, if researchers in this field had delved into the literature, or had corresponded with any scientist who had experience in the optimization of production of microbial metabolites in “pre-genomic times,” almost all of whom would have been from the antibiotic industry where publications were via patents, not scientific journals, then they would have found that in 1998, Strobel reported increasing the yield of taxol and maintaining it by using specific plant “extracts” to supplement the media (Li et al., 1998). Coming into the present, in a very nice example, the Oberlies group demonstrated that extracts from the leaves of the plant that “produced” the flavolignins silybin A and B (**Figure 4**; 26, 27), reversed the loss shown on subculturing by growing on a medium supplemented with autoclaved leaves of the nominal producing plant, *Silybum marianum* (El-Elimat et al., 2014). The previous year, the Proksch group in Germany published an excellent review on endophytes and their products, also pointing out the same phenomenon (Aly et al., 2013). This type of supplementation was used not only in plant symbiont studies as very recently, the Ilan group in Israel demonstrated that addition of sponge skeletons to a microbial culture increased the “richness” of arsenic-tolerant bacteria from *Theonella swinhoei* (Keren et al., 2016).

That endophytes are one of the producers (in some cases) is shown by the large amount of work performed over the years on the “actual source” of the well-studied “plant product,” maytansine (**Figure 4**; 28). From early days, its resemblance to a well-known series of bacterial metabolites led people to speculate on the actual source of the material, with one valid idea being conversion from the bacterial metabolite, ansamitocin P3, which differs only in an ester (**Figure 4**; 29) known to be produced in the rhizosphere of *Maytenus* species. This molecule would subsequently be taken up from the plant’s rhizosphere and modified to produce maytansine by transesterification. In recent excellent work by the Spiteller group, in a paper in 2014, working with a *Putterlickia* species (one of the first plants from which maytansine was isolated), demonstrated that microbes in the plant’s rhizosphere were the source of maytansine, without any plant involvement (Kusari et al., 2014).

In contrast, and quite unexpected, were the findings reported from the same group in their recent 2016 paper. Using a maytansine-producing *Maytenus serrata* plant from Cameroon, and following the biosynthesis of maytansine in this plant, they showed a very strong relationship between endogenous “as yet uncultivated microbes” and the production of the desired metabolite, but demonstrated that the required chlorination step definitively occurred in the rhizosphere microbes. However, an unexpected finding was that the starter unit, 3-amino-5-hydroxybenzoic acid (AHBA), was produced by both these microbes and the plant. So depending upon the geographic area and perhaps the genus and species of the “nominal producing plant,” the route to the same compound can differ (Kusari et al., 2016). From their earlier data, this was not the expected result, but the evidence is there.

These findings also imply the usage of quorum sensing in the interactions between not only the endophyte/rhizosphere, but also with the plant itself (Kusari et al., 2015). Thus findings such as these need to be considered in any evaluation of plant biotechnology as a route to metabolite production, as plant explants are not axenic.

That quorum sensing agents of various chemical classes, not only the simple compounds usually mentioned in the microbial literature, are used in these interactions was shown by other recent papers from the Spiteller group (Wang et al., 2015; Li et al., 2016a). The short review published by Ludwig-Muller (2015) appears to be quite prescient in light of these recent papers, and should be read in conjunction with the Spiteller group publications, not forgetting the earlier work by Strobel that really began this area of investigation as far as plants are concerned.

## Fungal–Bacterial Interactions

Though interactions on an organism to organism scale between fungi and bacteria are well known, there is one particular interaction that is most unusual, and that is where the toxins, normally considered to be fungal products, are in fact from a symbiotic bacterium in one structure and from a subsequent chemical modification by the host fungus in the other. In 2005 to 2006, the Hertweck group demonstrated that the “fungal toxin” rhizoxin (**Figure 4**; 30) was not produced by a *Rhizopus* fungal species, but from a symbiotic bacterium later identified as a *Burkholderia* species, and then demonstrated the variety of structures that could be obtained from culturing the bacterium outside of the fungus (Partida-Martinez and Hertweck, 2005, 2007; Scherlach et al., 2006; Partida-Martinez et al., 2007).

However, the story did not end there, as later work from the same group demonstrated that a much more involved process occurred. The bacterium actually produced a mono-epoxy derivative, WF-1360F (**Figure 4**; 31) originally thought to be an artifact, that was biologically active as an antitubulin agent, and then they found other *Rhizopus* species that only produced this mono-epoxy compound but contained the endophytic bacterium. By some very clever investigative work involving transfer of the bacterium between fungal species, in 2012 it was proven that the host fungus actually converts the mono-epoxy WF-1360F to the diepoxy derivative, rhizoxin, since the required P450 enzyme does not occur in all *Rhizopus* species (Scherlach et al., 2012). It should also be pointed out that both of these compounds are tubulin interactive agents in their own right.

Thus Nature has evolved a ternary production system, and similar systems may well be operative in this type of interaction in other organism complexes, though binary systems are more common. Examples can be found in the comprehensive review by Scherlach et al. (2013) Annual Reviews of Microbiology, and in the biosynthesis of other polyketides when a different *Burkholderia* species was co-cultured with *Rhizopus microspores* (Ross et al., 2014). Also in 2014, the Hertweck group published an article demonstrating how bacteria could enter fungi via an “active” process, thus answering how this symbiotic relationship could have occurred (Moebius et al., 2014). Obviously this is only the beginnings of the potential interaction(s) but it does demonstrate that what appears to be “gospel” often is not.

## Mimicking *In situ* Microbial Interactions

In the environment, irrespective of whether it is marine or terrestrial in nature, microbes from all kingdoms (Archaea, Prokarya, and Eukarya) are in close intimate contact with each other, as mentioned above in the discussion on maytansine, and chemical messages are exchanged between and within organisms from all kingdoms. These messages range from simple lactones to peptides and may be species specific or be capable of interacting across kingdoms.

It was realized some years ago, following inspection of the full genomes of fungi and bacteria, that there were many more potential BGCs than accounted for by the molecules that had been found from the “simple” fermentations that had been used for the previous 60 plus years. These “cryptic clusters” were sometimes found to be under some form of epigenetic control, so when small molecules that were demethylase or histone deacetylase (HDAC) inhibitors, were added to a single microbe fermentation, the “mixture” could produce molecules that had not previously been reported. A series of excellent papers demonstrating these techniques were published by the Cichewicz group at the University of Oklahoma in 2009–2010 and these should be consulted for this type of experimentation (Fisch et al., 2009; Henrikson et al., 2009; Cichewicz, 2010; Cichewicz et al., 2010).

Working with fungi, in 2006 the Keller group reported on the potential for genomic control in *Aspergillus* and demonstrated that contrary to the then “current dogma,” secondary metabolite biosynthetic clusters in fungi, or at a minimum in *Aspergillus*, are not randomly spread across chromosomes but are usually found as “groupings” on one or two very spatially close chromosomes (Bok et al., 2006). Very significant work on fungal secondary metabolite control has been performed by Keller and her associates over the last 10 years, demonstrating that there are well over 100 previously unrecognized secondary metabolite clusters in some species, and that exquisite control mechanisms are present, with their latest paper demonstrating that plant-like isoquinolines are present in *Aspergillus fumigatus* (Baccile et al., 2016).

In addition to looking for control via the use of epigenetic modulators, or using endogenous genetic controls, a simple system that mimics what happens in Nature, though starting with just two dissimilar microbes, has led to the identification of novel agents not seen on fermentation of the individual microbes in separate vessels.

Before giving the results of some of these more modern experiments, it is necessary to point out for the record, that back in the late 1960s to early 1970s, microbiologists were interested in what might occur if “signal systems” could be set up between individual microbes. These ideas led to a device known as the “EcoloGen” which was originally produced by New Brunswick Scientific in the early 1970s. Effectively, this was a central chamber with four cylindrical vessels coming off at 90° to each other, with the openings to the central vessel able to be blocked, or have specific ionic or molecular weight filters interposed between each individual side vessel and the central one. This would permit the cultivation of up to five different microbes and allow molecules to pass between them if the filter(s) permitted such transfer. The author used one of these devices in the pharmaceutical industry at that time, but the analytical systems were not refined enough, 40 plus years ago, to permit identification of “induced metabolites,” only that their new, or improved biological activities, could be measured as producers of “antibiotic activity.”

The publication by Martin in 1974 may have been the first to demonstrate the value of this instrument in studying effects on the cyanophyte *Gomphosphaeria aponina* when attempting to evaluate control of toxic blooms in fresh water (Martin et al., 1974). Then in 1978 there was a report from Canadian scientists commenting on the use of the EcoloGen to study the effects of antibiotic treatment on experimental mixed infections, perhaps a reversal of what is done today with mixed cultures (Lebrun et al., 1978). Finally, the last original publication that I can find, was another by Martin and Martin (1986), studying effects of growth inhibitors on *Hydrilla verticillata*, an invasive fresh water plant, though I have little doubt, due to my own experience, that many experiments in industry were never published in academic journals. Today, the modern technique is the use of diffusion chambers on a micro-scale to induce the fermentation of sponge-associated or other host bacteria, but though the investigators may not have been aware of the earlier methods, these effectively duplicated the earlier concept with newer methodologies (Steinert et al., 2014).

Perhaps the first formal reports of activity from designed experiments in co-culture were those in 1994 by Sonnenbichler et al. (1994) and then in Burgess et al. (1999). However, in both of these reports, only increased biological activity was reported. Then in 2001, the Fenical group at the Scripps Institution of Oceanography in California, reported the production of a new antibiotic, pestalone (**Figure 4**; 32) from challenging a marine fungus, *Pestalotia* sp., with a marine α-proteobacterium (Cueto et al., 2001). Further work with the same bacterium and the marine-derived fungus *Libertella* sp., yielded the new cytotoxic diterpenoids, libertellenones A–D (**Figure 4**; 33–36), reported in 2005 (Oh et al., 2005). In 2007, the same group reported the production of the cyclic depsipeptides the emericellamides A and B (**Figure 4**; 37, 38) when the marine-sourced fungus *Emericella* sp., was co-cultured with the obligate marine bacterium, *Salinispora arenicola* (Oh et al., 2007). Since that time, a number of novel metabolites have been reported in the literature with some representative examples being discussed in the preamble to an article by the Jaspars’ group in 2013, covering their findings on co-culture of *Aspergillus fumigatus* with the type strain of *Streptomyces bulli*, a bacterium from an hyper-arid soil (Rateb et al., 2013).

## Conclusion

In the last 2–3 years, there have been a significant number of papers published that give further information on how one may use the types of interactions discussed above, and most importantly, describe methodologies that can be used to “interrogate” the results. These include, but are not limited to: discussion of eliciting secondary metabolism in actinomycetes (Seyedsayamdost et al., 2012; Abdelmohsen et al., 2015; Rutledge and Challis, 2015); methodologies for determining the compounds expressed (Adnani et al., 2014; Luzzatto-Knaan et al., 2015; Medema and Fischbach, 2015; Mohimani and Pevzner, 2016); analyses of the biosynthetic clusters identified in fungi to published natural product structures (Li et al., 2016b); small-scale plate-based techniques for fungal co-culture (Bertrand et al., 2014); on-demand production of secondary metabolites (Bode et al., 2015); mixed culture of endophytes (Chagas et al., 2013); metabolomics in induced cultures (Derewacz et al., 2015; van der Lee and Medema, 2016; Zhang et al., 2016a,b; Ziemert et al., 2016); use of synthetic biological techniques to further expand the chemical biodiversity discovered (Smanski et al., 2016); and a recent review of the array of approaches to study such cryptic cluster expression by Zarins-Tutt et al. (2016).

Finally, there are three excellent papers on materials from symbioses in plants, marine organisms, and other animals that should be read by everyone interested in this topic. The first is a truly excellent compendium of material from plant and marine invertebrate symbioses (Florez et al., 2015), the second covering animal–microbe interactions including the major compounds from insect–microbe symbioses (Kieft and Simmons, 2015), whilst the third discusses the “utility” of such materials as signal molecules (Hillman and Goodrich-Blair, 2016).

## Author Contributions

The author confirms being the sole contributor of this work and approved it for publication.

## Conflict of Interest Statement

The author declares that the research was conducted in the absence of any commercial or financial relationships that could be construed as a potential conflict of interest.
